# Child maltreatment-related dissociation and its core mediation schemas in patients with borderline personality disorder

**DOI:** 10.1186/s12888-020-02797-5

**Published:** 2020-08-12

**Authors:** Mohsen Khosravi

**Affiliations:** Department of Psychiatry and Clinical Psychology, Baharan Psychiatric Hospital, Zahedan University of Medical Sciences, Zahedan, Postal Code: 9813913777 Iran

**Keywords:** Borderline personality disorder, Dissociation, Schema therapy, Child maltreatment

## Abstract

**Background:**

From a developmental and pathogenic perspective, child maltreatment is strongly linked to later dissociative symptoms, as ultimate forms of human response to chronic stress. The present study aimed to investigate the mediating role of early maladaptive schemas (EMSs) in the relationship between child maltreatment and dissociation among patients with borderline personality disorder (BPD).

**Methods:**

In this cross-sectional study, a total of 152 BPD patients (men: 52%; women: 48%) with an average age of 29.64 years (standard deviation (SD) = 7.29, range = 18–47) were selected by systematic random sampling from the patients who referred to Baharan psychiatric hospital in Zahedan, Iran, with the sampling interval of 3. The Childhood Trauma Questionnaire-Short Form, Dissociative Experiences Scale, and Young Schema Questionnaire-Short Form 3 were used to assess the patients. Data were analyzed using SPSS v25 software, and the statistical significance level was set at *p* < 0.05.

**Results:**

four main findings were obtained from the present study: (1) Heterogeneity of the levels of dissociation (LOD) in BPD patients; (2) The predicting roles of emotional neglect (EN), vulnerability to harm, and defectiveness/shame schemas in the total DES scores; (3) The vague role of childhood sexual abuse (SA) in developing dissociative symptoms; and (4) The mediating role of the core schemas of vulnerability to harm (β = 0.28, 95% confidence interval (CI): 0.04, 0.61) and defectiveness/shame (β = 0.21, 95% CI: 0.008, 0.45) in the relationship between EN and dissociation.

**Conclusions:**

Regarding the heterogeneity of LOD and its crucial role in the successful treatment of BPD patients, it is highly essential to evaluate the present-state dissociation of the patient during the diagnosis process and provide effective interventions to reduce it. The obtained results highlighted the potential role of schema therapy in reducing dissociative responses to emotional stimuli (based on EN), vulnerability to harm, and defectiveness/shame. Nevertheless, psychopathology of dissociation among BPD patients should be further investigated in depth.

## Background

Dissociation is a complex phenomenon with impacts on various areas, including memory, emotion, sensory perception, and cognition [1]. People with this problem may experience either positive symptoms (i.e., intrusive symptoms) or negative symptoms (i.e., apparent losses) [2]. Zanarini et al. [3] reported that dissociation scores in patients with borderline personality disorder (BPD) were higher compared to healthy and psychiatric controls. They presented three levels of dissociation (LOD), i.e., mild, moderate, and severe, and found these symptoms to be heterogeneous in origin. Some researchers, such as Liotti [4] and Meares [5], asserted that dissociation is a central factor in the development of trauma-related disorders. However, a recent meta-analysis of 2035 subjects [6] revealed that such a phenomenon occurs as an extreme form of experiential avoidance in BPD patients. In this regard, Korzekwa et al. [7] postulated that the severity of dissociation is typically correlated with the severity of child maltreatment and disruption of attachment. This assertion has been strongly supporting Janet’s ideas about early maltreatment and dissociation [8], arguing that caregivers with maltreating actions would negatively affect child’s attachment security, strategies for coping with stress, and the sense of self. This concept has further led to the development of the theory of Structural Dissociation of the Personality (SDP). SDP is characterized by the loss of integration between daily-life and defensive action systems, as a result of a continuous threat to bodily integrity and/or life [9]. Both these findings and clinical observations have almost assumed that dissociation, as a part of BPD symptomatic manifestation, is a form of human response to extreme stress at different levels among the patients [6].

Many studies have proved the relationship between BPD and child maltreatment [1] since the first attempt by Judith Herman [10]. The emotional abuse (EA) and emotional neglect (EN) experiences might cause gradual damage to beliefs about the self and develop maladaptive models of self, other, and self-in-relation to others. Accordingly, the investigation of internal cognitive processes as the mediating mechanism can help facilitate the recognition of EA- and EN-induced clinical symptoms [11]. These underlying assumptions are known as “relational schemas” or “core beliefs” in cognitive literature [12]. The schemas are the organized representations of subjects’ prior experiences that have impacted their perceptions, thoughts, and behavior [13]. Young et al. [13] designed a specific questionnaire for the assessment of early maladaptive schemas (EMSs) and divided them into unconditional beliefs about the self and conditional beliefs. Based on the unconditional maladaptive schemas, a change in individual behavior can never alter the outcome since the person is always recognized as incompetent, unlovable, unworthy, and bad. By contrast, conditional schemas imply that an individual has a hope of changing the outcome, even temporary, through subjugation, self-sacrifice, emotional inhibition, and unrelenting standards. Since an individual with experiences of childhood-EN suffers higher levels of distress through developing salient EMSs, child maltreatment is expected (based on a developmental and pathogenic perspective) to be strongly linked to later dissociative symptoms as ultimate forms of human response to chronic stress [1, 11, 14].

Based on the above reasoning, it could be concluded that the correction of the dissociation concept and optimization of its treatment entails a deep understanding of the dissociation-related processes. Accordingly, the current study aims to investigate the mediating role of EMSs in the relationship between child maltreatment and dissociative symptoms among borderline patients.

## Methods

### Participants

In this cross-sectional study, 152 BPD patients (men: 52%; women: 48%) were selected from the patients who referred to Baharan psychiatric hospital in Zahedan, Iran, via the systematic random sampling method with the sampling interval of 3. The average age of patients was 29.64 years (standard deviation (SD) = 7.29, range = 18–47) (see Table 1). The specified inclusion criteria were as follows: (1) Patients with Borderline Personality Inventory (BPI) scores higher than 10 and a confirmed diagnosis of BPD through the Structured Clinical Interview for DSM-5 Personality Disorders (SCID-5-PD) [15]; (2) Aged 18–50; (3) Ability to read, write, and understand. Also, the exclusion criteria comprised the followings: (1) Serious physical illness; (2) Brain traumatic injury; (3) Comorbidity of bipolar disorder; (4) Comorbidity of schizophrenia and other psychotic disorders; (5) Epileptic disorders; (6) Intellectual disability; (7) incomplete questionnaire.

**Table 1:** Demographic information among participants (*N* = 152)

Variables		M (SD; range)	N (%)
**Age**		29.64 (7.29; 18–47)	
**Gender**	Male		79 (52.0)
	Female		73 (48.0)
**Marital status**	Single		64 (42.1)
	Married		71 (46.7)
	Divorced		17 (11.2)
**Level of education**	Non-degree		125 (82.2)
	High school diploma		15 (9.9)
	Academic degree		12 (7.9)
**Income**	< 1,000,000 Rials		135 (88.8)
	≥ 1,000,000 Rials		17 (11.2)

### Procedure

After obtaining the approval of the current research project from the Research Medical Center School of Zahedan University and the prior permission from the relevant Ethics Committee with IR.ZAUMS.REC.1397.451 code of ethics, the subjects were given the consent forms to sign. The study was performed in compliance with the declaration of Helsinki, i.e., subjects were told that their participation was optional and they could leave the study for any reason. After obtaining their consent, patients were given the Childhood Trauma Questionnaire-Short Form (CTQ-SF), Dissociative Experiences Scale (DES), and Young Schema Questionnaire-Short Form 3 (YSQ-S3). The questionnaires were anonymous to keep the personal information of the patients completely confidential.

### Measures

#### BPI

A Persian version of BPI was used in screening for BPD, which involves a 53-item yes-or-no questionnaire concerning four factors, namely (1) identity diffusion, (2) primitive defense mechanisms, (3) impaired reality testing, and (4) fear of closeness. A cut-off score above 10 for 20 items of the questionnaire points out that the person is more likely to have BPD. According to the study conducted by Mohammadzadeh and Rezaei [16], the test-retest reliability coefficient of this questionnaire was 0.80, and the internal consistency reliability coefficients for Factors 1 to 4 were 0.63, 0.74, 0.66, and 0.62, respectively. Also, the Persian version of BPI exhibited acceptable convergent validity against the Borderline Personality Scale (*r* = 0.70). In this study, the Cronbach’s alpha coefficients for the BPI subscales were ranged from 0.75 to 0.88.

#### SCID-5-PD

A confirmed diagnosis of BPD was performed through SCID-5-PD. The questionnaire was a semi-structured diagnostic interview to be used by clinicians and researchers to assess the 10 DSM-5 personality disorders across clusters A, B, and C as well as other specified personality disorders. Various research reports have validated the reliability of SCID-5-PD [15].

#### DES

A Persian version of DES was utilized to evaluate dissociation symptoms. In this 28-item questionnaire, the respondents were asked to rate their experiences for each item on a scale of 0 to 100% (i.e., ranging from “never” to “always”). Total scores were calculated by averaging the 28 item scores. Also, there are three LODs, namely mild (DES scores < 10), moderate (DES scores = 10–29.9), and severe (DES scores ≥30). Sajadi et al. [17] demonstrated that the peritraumatic and persistent DESs have excellent homogeneity (Cronbach’s alpha = 0.87 and 0.91, respectively) and very high consistency over time (intraclass correlation coefficient = 0.87). Also, the correlation coefficient between peritraumatic and persistent DES scores was 0.81. Moreover, the Pearson correlation analysis revealed a significant positive correlation between DES (peritraumatic and persistent) and posttraumatic stress disorder (PTSD) scores at the level of 0.05 (*r* = 0.38 and 0.55, respectively). In this study, the Cronbach’s alpha coefficient for the total scale was 0.90.

#### YSQ-S3

Participants’ EMSs were measured using a Persian version of the YSQ-S3 questionnaire, including 90 items, each with a 6-point Likert scale (1 = entirely untrue of me, 6 = describes me perfectly). This instrument was designed by Young to assess 18 schemas, including abandonment, social isolation/alienation, pessimism, emotional inhibition, dependence, approval/recognition-seeking, emotional deprivation, vulnerability to harm, enmeshment, subjugation, unrelenting standards, self-punitiveness, defectiveness/shame, mistrust/abuse, failure, self-sacrifice, entitlement, and insufficient self-control. Continuous variables for each schema were created by averaging the participants’ ratings over the assessment items. As each subscale consisted of 5 items, the score obtained on the subscales varied between 5 and 30. Yousefi and Shirbagi [18] indicated a high level of Cronbach’s alpha and split-half (86 and 91%, respectively) reliability coefficients of the YSQ-S3 and the extracted factors. Also, the convergent and discriminate validity of the YSQ-S3 was examined by some measurement tools in terms of psychological distress, positive and negative affect, self-confidence, cognitive vulnerability to depression, and personality disorder. The correlation results for the six former criteria were sequentially as follows: 37, 34, 40, 39, 35, and 36%, which were significant at *p* < 0.001. In this study, the Cronbach’s alpha coefficients for the YSQ-S3 subscales were ranged from 0.67 to 0.92.

#### CTQ-SF

A Persian version of CTQ-SF was employed for the measurement of child maltreatment, consisting of 28 items scored on a 5-point Likert scale. The questionnaire was developed to measure five subtypes of child maltreatment, namely EA, physical abuse (PA), sexual abuse (SA), EN, and physical neglect (PN). According to the study of Garrusi and Nakhaee [19], the test-retest reliability coefficient of this questionnaire was 0.90, and the internal consistency reliability coefficients for the four subscales were 0.86, .0.85, 0.84, and 0.60, respectively, with a mean of 0.79. Also, the Persian version of CTQ-SF exhibited acceptable convergent validity against the 28-items General Health Questionnaire (*r* = 0.40). In this study, the Cronbach’s alpha coefficients for the CTQ-SF subscales were ranged from 0.75 to 0.90.

### Data analysis

Descriptive statistical methods, including mean and standard deviation, were applied for data analysis. Pearson correlation coefficient, Spearman’s ρ, and point-biserial correlation coefficient were performed to assess the correlation between study variables. The mediating role of EMSs in the relationship between child maltreatment and dissociation was tested through the Hayes’ Process v3.3 macro in SPSS [20]. As outlined in Preacher and Hayes [21], mediation emerges when the indirect effect is significant and the confidence intervals exclude zero. Data were analyzed using SPSS v25 software, and the statistical significance level was set at *p* < 0.05.

## Results

### Preliminary analysis

**Fig. 1:**
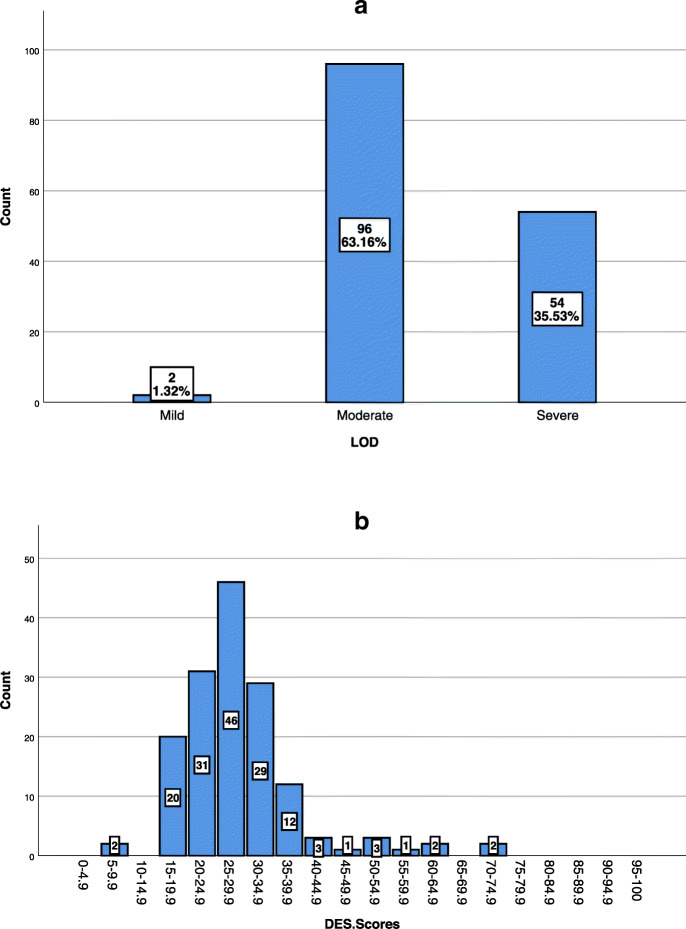
**a** Levels of dissociation (LOD) including mild, moderate, and severe dissociative symptoms among patients with borderline personality disorder; **b** The distribution of mean DES scores for patients with borderline personality disorder. ***Note.*** Mild (DES scores < 10); Moderate (DES scores = 10–29.9); Severe (DES scores ≥30)

Figure 1(a) and Fig. 1(b) illustrate the LOD and distribution of mean DES scores among BPD patients, respectively. Based on Fig. 1(a), about 1.32% of the patients had a mild LOD, which was generally considered to be in the low range. About 63.16% of the patients had moderate LOD, which is commonly observed in a number of non-trauma-related psychiatric disorders such as feeding and eating disorders. The remaining 35.53% of the patients had severe LOD, which is frequently seen in patients with PTSD or dissociative disorders [3].

According to Table 2, dissociation was positively correlated with PA (*r* = 0.21, *p* < 0.01), EA (*r* = 0.31, *p* < 0.01), PN (*r* = 0.24, *p* < 0.01), EN (*r* = 0.52, *p* < 0.01), abandonment (*r* = 0.19, *p* < 0.01), pessimism (*r* = 0.34, *p* < 0.01), approval/recognition-seeking (*r* = 0.27, *p* < 0.01), vulnerability to harm (*r* = 0.59, *p* < 0.01), enmeshment (*r* = 0.18, *p* < 0.05), subjugation (*r* = 0.21, *p* < 0.01), unrelenting standards (*r* = 0.23, *p* < 0.01), self-punitiveness (*r* = 0.29, *p* < 0.01), defectiveness/shame (*r* = 0.57, *p* < 0.01), entitlement (*r* = 0.26, *p* < 0.01), and borderline personality symptomatology (BPS) (*r* = 0.37, *p* < 0.01). Moreover, LOD was positively correlated with PA (*r* = 0.18, *p* < 0.05), EA (*r* = 0.27, *p* < 0.01), EN (*r* = 0.36, *p* < 0.01), and BPS (*r* = 0.30, *p* < 0.01). Furthermore, EA was positively correlated with BPS (*r* = 0.19, *p* < 0.05). Significant correlation between gender and dissociation (*r* = 0.18, *p* < 0.05) and between level of education and dissociation (*r* = 0.17, *p* < 0.05) were seen.

**Table 2:** Correlations of study variables (*N* = 152)

Variables	SA	PA	EA	PN	EN	BPS	DES^**1**^	LOD^**2**^
**Abandonment**	−0.12	− 0.18^*^	− 0.03	− 0.17^*^	0.15	0.26^**^	0.19^*^	0.12
**Social isolation/alienation**	−0.02	− 0.03	0.02	− 0.01	0.00	0.12	0.09	0.07
**Pessimism**	−0.11	−0.13	0.12	−0.06	0.24^**^	0.21^**^	0.34^**^	0.19^*^
**Emotional inhibition**	−0.06	−0.01	− 0.06	−0.19^*^	− 0.13	0.03	0.08	0.11
**Dependence**	−0.09	−0.25^**^	− 0.10	−0.16^*^	− 0.15	0.14	− 0.00	0.01
**Approval/recognition-seeking**	−0.20^*^	0.17^*^	0.14	−0.10	0.24^**^	0.14	0.27^**^	0.22^**^
**Emotional deprivation**	−0.04	− 0.15	−0.05	− 0.12	0.03	0.22^**^	0.08	0.07
**Vulnerability to harm**	−0.13	0.09	0.19	0.01	0.35^**^	0.24^**^	0.59^**^	0.50^**^
**Enmeshment**	−0.19^*^	−0.12	−0.06	− 0.14	0.01	0.14	0.18^*^	−0.00
**Subjugation**	−0.06	−0.16^*^	− 0.01	−0.08	0.00	0.15	0.21^**^	0.11
**Unrelenting standards**	−0.19^*^	−0.12	− 0.09	−0.24^**^	0.04	0.10	0.23^**^	0.16^*^
**Self-punitiveness**	−0.25^**^	0.01	−0.07	−0.01	0.08	0.10	0.29^**^	0.18^*^
**Defectiveness/shame**	−0.11	0.08	0.12	0.01	0.35^**^	0.22^**^	0.57^**^	0.43^**^
**Mistrust/abuse**	−0.08	−0.01	0.05	−0.04	0.10	0.13	0.04	0.00
**Failure**	−0.07	− 0.21^**^	−0.00	− 0.20^*^	−0.02	0.09	−0.03	− 0.09
**Self-sacrifice**	−0.08	0.07	0.13	0.07	0.13	0.03	0.12	0.03
**Entitlement**	−0.24^**^	− 0.04	0.01	− 0.18^*^	0.13	0.13	0.26^**^	0.20^*^
**Insufficient self-control**	−0.26^**^	−0.11	0.12	−0.22^**^	0.16^*^	0.19^*^	0.11	0.04
**BPS**	0.01	0.08	0.18^*^	0.11	0.27^**^	1.00	0.37^**^	0.30^**^
**DES**^**1**^	0.00	0.21^**^	0.31^**^	0.24^**^	0.52^**^	0.37^**^	1.00	0.83^**^
**LOD**^**2**^	0.02	0.18^*^	0.27^**^	0.14	0.36^**^	0.30^**^	0.83^**^	1.00

***Note.*** *BPS* Borderline personality symptomatology; *DES* Dissociative Experiences Scale; *EA* Emotional abuse; *EN*: Emotional neglect; *LOD* Levels of dissociation; *PA* Physical abuse; *PN* Physical neglect; *SA* Sexual abuse

***Note.*** ^1^Total score; ^2^Mild (DES scores < 10); Moderate (DES scores = 10–29.9); Severe (DES scores ≥30)

***Note.*** ^*^p < 0.05; ^**^p < 0.01

### Predictor variables of dissociation in BPD patients

Model summary of multiple regression analysis to evaluate the predictor variables of dissociation in BPD patients are presented in Table 3. As shown, EN, vulnerability to harm, and defectiveness/shame accounted for the predictor variables for a significant proportion of the variance in the total DES scores.

**Table 3:** Model summary of multiple regression analysis to evaluate the predictor variables of dissociation in patients with borderline personality disorder (*N* = 152)

Predictors	β	SE	t	95% CI
**Gender**	0.38	1.27	0.30	−2.12, 2.89
**Degree level**	−0.04	1.14	−0.03	−2.31, 2.22
**PA**	−0.17	0.25	−0.69	−0.68, 0.32
**EA**	0.07	0.05	1.32	−0.03, 0.18
**PN**	0.21	0.24	0.86	−0.27, 0.69
**EN**	0.63	0.19	3.21^**^	0.24, 1.02
**BPS**	0.44	0.22	1.98^*^	0.001, 0.88
**Abandonment**	−0.09	0.16	−0.58	−0.41, 0.22
**Pessimism**	−0.21	0.19	−1.08	−0.61, 0.17
**Approval/recognition-seeking**	−0.17	0.16	−1.10	−0.49, 0.14
**Vulnerability to harm**	0.94	0.26	3.61^***^	0.42, 1.46
**Enmeshment**	0.05	0.18	0.29	−0.30, 0.41
**Subjugation**	0.34	0.19	1.74	−0.04, 0.73
**Unrelenting standards**	0.16	0.20	0.79	−0.24, 0.57
**Self-punitiveness**	0.25	0.19	1.31	−0.12, 0.63
**Defectiveness/shame**	0.65	0.24	2.66^**^	0.16, 1.13
**Entitlement**	−0.26	0.20	−1.28	−0.66, 0.14
**R**	0.75			
**R**^**2**^	0.57			
**F (df1, df2)**	10.50 (17, 134)^***^			

***Note.*** *BPS* Borderline personality symptomatology; *EA* Emotional abuse; *EN* Emotional neglect; *PA* Physical abuse; *PN* Physical neglect; *SA* Sexual abuse

***Note.*** ^*^p < 0.05; ^**^p < 0.01; ^***^p < 0.001

### Mediation analysis

**Fig. 2:**
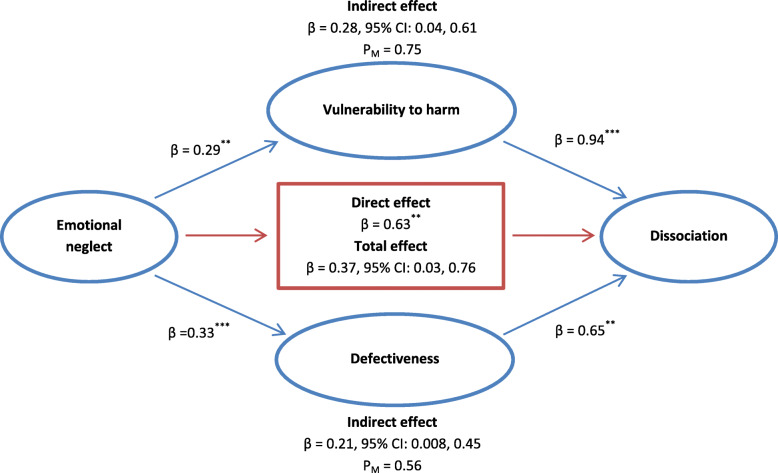
Illustration of the results of the mediation analysis described in the text, which tested vulnerability to harm and defectiveness/shame as the potential mediators of the relationship between emotional neglect and dissociation by controlling for sociodemographic variables (included gender and level of education), Borderline personality symptomatology (BPS), physical abuse (PA), emotional abuse (EA), and physical neglect (EN) in patients with borderline personality disorder (*n* = 152). ***Note.*** P_M_: Effect size (ratio of indirect to total effect). ***Note.*** ^*^p < 0.05; ^**^p < 0.01; ^***^p < 0.001

The mediation analysis was performed using Hayes’ process tool in SPSS (model = 4, bootstrap samples = 5000). As hypothesized, a significant indirect effect of EN was distinctly observed on dissociation through vulnerability to harm and defectiveness/shame (β = 0.28, 95% confidence interval (CI): 0.04, 0.61; β = 0.21, 95% CI: 0.008, 0.45, respectively). The mediators (i.e., vulnerability to harm and defectiveness/shame) could account for roughly 75% (P_M_ = 0.75) and 56% (P_M_ = 0.56) of the total effect of EN on dissociation, respectively. Hence, the overall hypothesis that vulnerability to harm and defectiveness/shame mediate the effect of EN on dissociation was supported (see Fig. 2).

## Discussion

The findings of the present study can be divided into four major parts. As the first part, 1.32% of BPD patients had a mild LOD, 63.16% had a moderate LOD, and 35.53% had a severe LOD, similar to that reported for patients with PTSD or dissociative disorders. These distributions are inconsistent with the results of the study performed by Zanarini et al. [3], wherein the LOD in BPD patients were 31.7, 42.1, and 26.2% for mild, moderate, and severe modes, respectively. Besides, the heterogeneity of the LOD (see Fig. 1(b)) was consistent with the results obtained by Zanarini et al. [3] and contradicted the findings of previous studies [22–25]. The earlier investigations as a whole demonstrated that the majority of BPD patients had a relatively high LOD that was not different from the LOD in patients with trauma-related disorders [22–27]. By contrast, the results of the present study and Zanarini et al. [3] showed that a large number of BPD patients had moderate LOD. This result regarding the heterogeneity of dissociative experiences might offer critical clinical implications. For instance, clinicians need to carefully evaluate the LOD in their BPD patients, instead of the assumption of severe LOD in all BPD patients [3]. This individualized method can also be applied to the selection of both the pharmacologic intervention and psychotherapeutic strategy. Indeed, BPD patients with a mild to moderate LOD may not have the same needs and demands as what BPD patients with severe LOD have [3].

As the second main finding of the present study, EN was able to predict a significant proportion of the variance in the total DES scores. This finding was consistent with the results obtained by Watson et al. [28], Van Den Bosch et al. [29], Simeon et al. [30], and Sar et al. [31], who identified EN as a predictor of dissociation. These results supported the hypothesis that EN engenders dissociative symptoms later in life. In detail, Bowlby’s attachment theory suggests that children make internal working models of attachment figures, of the self, and of self-in-relation to others based on their relationship with primary caregivers [32]. Thus, individuals who experienced EN may build maladaptive models of self, other, and self-in-relation to others by undermining beliefs about the self (an example for EN: “I am not entitled to anyone’s attention”) [33]. These maladaptive interpersonal expectations can lead to later adaptation (i.e., dissociative symptoms) by creating psychological distress [11].

As the third main finding of the present study, there was no association between SA and dissociative symptomatology among BPD patients. This result was consistent with findings by Zweig-Frank et al. [23, 24], Watson et al. [28], Simeon et al. [30], and Sar et al. [31], whereas it was inconsistent with the results obtained by Shearer [22], Brodsky et al. [27], and Van Den Bosch et al. [29], who announced childhood SA was a risk factor for dissociative symptomatology. However, further investigations are needed to reconcile these contradictory findings.

The fourth main result of the present study was that the vulnerability to harm and defectiveness/shame schemas played a mediating role in the relationship between EN and dissociation. This finding supported the role of dissociation in BPD proposed by Young et al. [13] and the key roles of EN and vulnerability to harm and defectiveness/shame in developing dissociative symptoms [34]. In other words, defectiveness/shame is an aversive affective experience caused by a very negative self-evaluation [35]. Individuals experiencing defectiveness/shame schemas often tend to hide the fact, whereas they attempt to push away the memories of childhood abuse through dissociation. However, dissociation by hiding the emotional and cognitive processes of traumatic experiences can disrupt the individual’s ability to recover from such sufferings [34, 35]. Additionally, in this study, the core schema of vulnerability to harm was identified as an unconditional maladaptive schema. This schema represents the importance of fear and helplessness as a lasting consequence of EN since the individual fails to prevent catastrophes and fears to re-experience them over and over in life. Having such beliefs and expectancy in life may further provoke a person to feel helpless and anxious and gradually develop more pervasive negative cognitive styles [35]. Children experiencing EN in stressful situations may not show their stress in future events to prevent emotional arousal in themselves. This mechanism may be enduring and encourage dissociation (i.e., a stress response) in children as a path for insulation against frightening or helplessness experiences. However, this behavioral practice in children may gradually lead them to use ineffective coping strategies [34]. These findings concerning the development of dissociation may have important clinical implications. For instance, dissociation prevents individuals from learning and reduces the effectiveness of learning-based therapies (e.g., dialectical behavior therapy) by inducing a disintegration of perception, consciousness, identity, and memory in individuals. Since both basic learning processes and classical conditioning follow a similar mechanism, the presence of any dissociative symptom in an individual prevents the person from acquiring any differential conditioning response. Therefore, based on the obtained results, schema therapy as a third-wave treatment may be effective in reducing dissociative responses to emotional stimuli (based on EN), vulnerability to harm, and defectiveness/shame. This therapy has aimed to reorganize these inner structures in BPD patients through four core mechanisms of change, including (a) limited reparenting, (b) experiential imagery and dialogue work, (c) cognitive restructuring and education, and (d) behavioral pattern-breaking [36].

The present study has suffered some limitations. First, the ability to generalize the findings to different cases has not been practical due to the small sample size and the selection of participants from one geographical area. Second, cross-sectional studies mostly fail to specify a definite reason behind a correlation. In detail, this limitation can prevent a deep understanding of the nature of the causal relationship between BPS and dissociation. The use of self-report measures is the third limitation since the obtained information may only reflect the feelings of patients during the assessment and not reveal the real emotions they have suffered. Thus, it is recommended that future studies focus on methodological limitations, including sole reliance on self-report measures due to the memory bias and demand characteristics, lack of empirical data, and the absence of ethnic differences.

Despite these limitations, the investigation could improve better comprehension of the potential mechanisms whereby abusive childhood experiences might impact the functioning of BPD patients. It is probably very imperative to understand these internalized representations for developing personality-targeted interventions and preventive approaches among victims of child maltreatment.

## Conclusions

In summary, four main findings emerge from the present study: (1) Heterogeneity of the LOD in BPD patients; (2) The predicting roles of EN, BPS, vulnerability to harm, and defectiveness/shame schemas in the total DES scores; (3) The vague role of childhood SA in developing dissociative symptoms; and (4) The mediating role of vulnerability to harm and defectiveness/shame schemas in the relationship between EN and dissociation. These findings have highlighted the importance of early interventions for the individuals who have developed internal working models of the self as worthless, others as abusive, or the world as threatening. Given the heterogeneous nature of LOD and its vital role in the success of treating BPD patients, it is critically needed to assess the present-state dissociation of the patient during the diagnosis and facilitate practical interventions to decrease it. The obtained results revealed the influential role of schema therapy in diminishing dissociative responses to emotional stimuli (based on EN), vulnerability to harm, and defectiveness/shame. Nevertheless, psychopathology of dissociation among BPD patients should be further investigated in depth.

## Data Availability

The datasets generated and analyzed during the current study are not publicly available because no consent for making the data publicly available was collected from the participants. However, the data are available from the corresponding author on reasonable request.

## Abbreviations

BPD: Borderline personality disorder
BPI: Borderline Personality Inventory
BPS: Borderline personality symptomatology
CTQ-SF: Childhood Trauma Questionnaire-Short Form
DES: Dissociative Experiences Scale
EA: Emotional abuse
EMSs: Early maladaptive schemas
EN: Emotional neglect
LOD: Levels of dissociation
PA: Physical abuse
PN: Physical neglect
PTSD: Posttraumatic stress disorder
SA: Sexual abuse
SCID-5-PD: Structured Clinical Interview for DSM-5 Personality Disorders
YSQ-S3: Young Schema Questionnaire-Short Form 3

## References

[1] Vermetten E, Spiegel D. Trauma and dissociation: implications for borderline personality disorder. Curr Psychiatry Rep. 2014;16(2):434. 10.1007/s11920-013-0434-8. 10.1007/s11920-013-0434-8 24442670

[2] Van Dijke A, van der Hart O, Ford JD, van Son M, van der Heijden P, Bühring M. Affect dysregulation and dissociation in borderline personality disorder and somatoform disorder: differentiating inhibitory and excitatory experiencing states. J Trauma Dissociation. 2010;11(4):424–43. 10.1080/15299732.2010.496140. 10.1080/15299732.2010.496140 20938867

[3] Zanarini MC, Ruser T, Frankenburg FR, Hennen J. The dissociative experiences of borderline patients. Compr Psychiatry. 2000;41(3):223–7. 10.1016/S0010-440X(00)90051-8. 10.1016/S0010-440X(00)90051-8 10834632

[4] Liotti G. Trauma, dissociation, and disorganized attachment: three strands of a single braid. Psychother: Theory Res Pract Train. 2004;41(4):472. 10.1037/0033-3204.41.4.472.

[5] Meares R. A dissociation model of borderline personality disorder (Norton series on interpersonal neurobiology). New York: Norton; 2012.

[6] Scalabrini A, Cavicchioli M, Fossati A, Maffei C. The extent of dissociation in borderline personality disorder: a meta-analytic review. J Trauma Dissociation. 2017;18(4):522–43. 10.1080/15299732.2016.1240738. 10.1080/15299732.2016.1240738 27681284

[7] Korzekwa MI, Dell PF, Pain C. Dissociation and borderline personality disorder: an update for clinicians. Curr Psychiatry Rep. 2009;11(1):82–8. 10.1007/s11920-009-0013-1. 10.1007/s11920-009-0013-1 19187714

[8] Janet P. The major symptoms of hysteria: fifteen lectures given in the medical school of Harvard University (between the fifteenth of October and the end of November, 1906). New York: Macmillan; 1907.

[9] Van der Hart O, Nijenhuis E, Steele K, Brown D. Trauma-related dissociation: conceptual clarity lost and found. Aust N Z J Psychiatry. 2004;38(11–12):906–14. 10.1080/j.1440-1614.2004.01480.x. 10.1080/j.1440-1614.2004.01480.x 15555024

[10] Herman JL, Perry JC, Van der Kolk BA. Childhood trauma in borderline personality disorder. Am J Psychiatry. 1989;146(4):490–5. 10.1176/ajp.146.4.490. 10.1176/ajp.146.4.490 2929750

[11] Wright MO, Crawford E, Del Castillo D. Childhood emotional maltreatment and later psychological distress among college students: the mediating role of maladaptive schemas. Child Abuse Negl. 2009;33(1):59–68. 10.1016/j.chiabu.2008.12.007. 10.1016/j.chiabu.2008.12.007 19167067

[12] Baldwin MW. Relational schemas and the processing of social information. Psychol Bull. 1992;112(3):461. 10.1037/0033-2909.112.3.461.

[13] Young JE, Klosko JS, Weishaar ME. Schema therapy: a practitioner’s guide. New York: Guilford Press; 2003.

[14] Egeland B, Susman-Stillman A. Dissociation as a mediator of child abuse across generations. Child Abuse Negl. 1996;20(11):1123–32. 10.1016/0145-2134(96)00102-0. 10.1016/0145-2134(96)00102-0 8958463

[15] First MB, Williams JB, Benjamin LS, Spitzer RL. User’s guide for the SCID-5-PD (structured clinical interview for DSM-5 personality disorder). Arlington: American Psychiatric Association; 2015.

[16] Mohammadzadeh A, Rezaei A. Validation of the borderline personality inventory in Iran. Int J Behavl Sci. 2011;5(3):269–77.

[17] Sajadi SF, Zargar Y, Honarmand MM, Arshadi N. Designing and testing a model of some precedents and outcomes of borderline personality disorder in high school students of Shiraz. Int J Sch Health. 2015;2(3):1–8. 10.17795/intjsh-26742.

[18] Yousefi N, Shirbagi N. Validating the young early maladaptive schema questionnaire (YEMSQ) among students. Iran J Psychiatry Behav Sci. 2010;4(1):38–46.

[19] Garrusi B, Nakhaee N. Validity and reliability of a Persian version of the childhood trauma questionnaire. Psychol Rep. 2009;104(2):509–16. 10.2466/PR0.104.2.509-516. 10.2466/PR0.104.2.509-516 19610481

[20] Hayes AF. Introduction to mediation, moderation, and conditional process analysis: a regression-based approach. New York: Guilford Press; 2017.

[21] Preacher KJ, Hayes AF. SPSS and SAS procedures for estimating indirect effects in simple mediation models. Behav Res Methods Instrum Comput. 2004;36(4):717–31. 10.3758/bf03206553. 10.3758/bf03206553 15641418

[22] Shearer SL. Dissociative phenomena in women with borderline personality disorder. Am J Psychiatry. 1994;151(9):1324–8. 10.1176/ajp.151.9.1324. 10.1176/ajp.151.9.1324 8067488

[23] Zweig-Frank H, Paris J, Guzder J. Dissociation in female patients with borderline and non-borderline personality disorders. J Personal Disord. 1994;8(3):203–9. 10.1521/pedi.1994.8.3.203.

[24] Zweig-Frank I-I, Paris J, Guzder if. Dissociation in male patients with borderline and non-borderline personality disorders. J Personal Disord. 1994;8(3):210–8. 10.1521/pedi.1994.8.3.210.

[25] Zweig-Frank H, Paris J, Guzder J. Psychological risk factors for dissociation and self-mutilation in female patients with borderline personality disorder. Can J Psychiatr. 1994;39(5):259–64. 10.1177/070674379403900504. 10.1177/070674379403900504 8044740

[26] Zweig-Frank H, Paris J, Guzder J. Psychological risk factors and self-mutiliation in male patients with BPD. Can J Psychiatr. 1994;39(5):266–8. 10.1177/070674379403900505. 10.1177/070674379403900505 8044741

[27] Brodsky BS, Cloitre M, Dnlit RA. Relationship of dissociation to self-mutilation and childhood abuse in borderline personality disorder. Am J Psychiatry. 1995;152(12):1788–92. 10.1176/ajp.152.12.1788. 10.1176/ajp.152.12.1788 8526247

[28] Watson S, Chilton R, Fairchild H, Whewell P. Association between childhood trauma and dissociation among patients with borderline personality disorder. Aust N Z J Psychiatry. 2006;40(5):478–81. 10.1080/j.1440-1614.2006.01825.x. 10.1080/j.1440-1614.2006.01825.x 16683975

[29] Van Den Bosch LM, Verheul R, Langeland W, Van Den Brink W. Trauma, dissociation, and posttraumatic stress disorder in female borderline patients with and without substance abuse problems. Aust N Z J Psychiatry. 2003;37(5):549–55. 10.1046/j.1440-1614.2003.01199.x. 10.1046/j.1440-1614.2003.01199.x 14511082

[30] Simeon D, Nelson D, Elias R, Greenberg J, Hollander E. Relationship of personality to dissociation and childhood trauma in borderline personality disorder. CNS Spectr. 2003;8(10):755–62. 10.1017/s109285290001912x. 10.1017/s109285290001912x 14712173

[31] Sar V, Akyuz G, Kugu N, Ozturk E, Ertem-Vehid H. Axis I dissociative disorder comorbidity in borderline personality disorder and reports of childhood trauma. J Clin Psychiatry. 2006;67(10):1583–90. 10.4088/jcp.v67n1014. 10.4088/jcp.v67n1014 17107251

[32] Waldinger RJ, Toth SL, Gerber A. Maltreatment and internal representations of relationships: Core relationship themes in the narratives of abused and neglected preschoolers. Soc Dev. 2001;10(1):41–58. 10.1111/1467-9507.00147.

[33] Kleindienst N, Limberger MF, Ebner-Priemer UW, et al. Dissociation predicts poor response to dialectial behavioral therapy in female patients with borderline personality disorder. J Personal Disord. 2011;25(4):432–47. 10.1521/pedi.2011.25.4.432. 10.1521/pedi.2011.25.4.432 21838560

[34] Feiring C. Emotional development, shame, and adaptation to child maltreatment. Child Maltreat. 2005;10(4):307–10. 10.1177/1077559505281307. 10.1177/1077559505281307 16252437

[35] Talbot JA, Talbot NL, Tu X. Shame-proneness as a diathesis for dissociation in women with histories of childhood sexual abuse. J Trauma Stress. 2004;17(5):445–8. 10.1023/B:JOTS.0000048959.29766.ae. 15633925

[36] Kellogg SH, Young JE. Schema therapy for borderline personality disorder. J Clin Psychol. 2006;62(4):445–58. 10.1002/jclp.20240. 10.1002/jclp.20240 16470629

